# Audiovisual Distraction During Mitral Transcatheter Edge-to-Edge Repair: A Sedation-Sparing Randomized Trial

**DOI:** 10.1016/j.jacadv.2026.102835

**Published:** 2026-05-21

**Authors:** Elias Rawish, Mira John, Felicitas Lemmer, Anke Constantz, Florian Genske, Christoph Marquetand, Hannes Alessandrini, Mulham Alhagi, Kira Erber, Patrick Kellner, Roza Saraei, Thomas Stiermaier, Ingo Eitel, Christian Frerker, Tobias Schmidt

**Affiliations:** a University of Luebeck, Medical Clinic II, University Hospital Schleswig-Holstein, Lübeck, Germany; b DZHK (German Centre for Cardiovascular Research), Partner Site North, Lübeck, Germany; c University Heart Center Lübeck, Lübeck, Germany; d University of Luebeck, Emergency Department, University Hospital Schleswig-Holstein, Lübeck, Germany; e University of Luebeck, Department of Anaesthesia and Intensive Care Medicine, University Hospital Schleswig-Holstein, Lübeck, Germany; f Department of Cardiology, Asklepios Westklinikum Hamburg, Hamburg, Germany

**Keywords:** M-TEER, mitral transcatheter edge-to-edge repair, patient satisfaction, propofol, structural heart intervention, transesophageal echocardiography

## Abstract

**Background:**

Conscious sedation is increasingly used for mitral transcatheter edge-to-edge repair (M-TEER), but balancing patient comfort with sedative-related hemodynamic and neurocognitive risk remains challenging in frail older adults.

**Objectives:**

We investigated whether audiovisual distraction (AVD) with video glasses reduces propofol requirements and improves patient experience during M-TEER performed under conscious sedation.

**Methods:**

In this prospective randomized controlled trial, 60 patients undergoing M-TEER were assigned 1:1 to AVD (video glasses with nature documentaries and headphones) or sham control (identical inactive device). Propofol-based sedation was managed according to a uniform blinded protocol using a pragmatic Richmond Agitation-Sedation Scale target of −2 to −3. The primary endpoint was the mean propofol dose (mg/kg/h). Secondary endpoints included norepinephrine use, anxiety and pain on numerical rating scales, overall patient satisfaction (measured by the German adaptation of the Client Satisfaction Questionnaire), and in-hospital outcomes.

**Results:**

The mean propofol dose was lower with AVD than control (4.6 ± 1.6 vs 5.9 ± 1.4 mg/kg/h; *P* = 0.002). Norepinephrine use was numerically lower (0.5 ± 1.1 vs 1.7 ± 3.4 μg/kg/h; *P* = 0.08). Procedural duration, hemodynamic fluctuations, and hospital length of stay were similar. AVD reduced postprocedural anxiety (1.1 ± 1.0 vs 2.5 ± 1.7; *P* = 0.001) and improved satisfaction (24.7 ± 3.0 vs 22.5 ± 2.8; *P* = 0.005). Safety events, including bleeding, pneumonia, conversion to general anesthesia, and clinically documented delirium, were comparable.

**Conclusions:**

AVD with video glasses is a safe, pragmatic adjunct to conscious sedation in M-TEER that reduces propofol requirements and improves patient-reported comfort and satisfaction without compromising procedural efficiency or in-hospital safety. (Does audiovisual distraction reduce the need for analgesic sedation and increase patient satisfaction in patients undergoing mitral valve clipping? A randomized controlled trial; DRKS00037370)

Mitral transcatheter edge-to-edge repair (M-TEER) is an established therapy for patients with moderate to severe symptomatic mitral valve regurgitation at high operative risk.^1^ It is increasingly performed under conscious sedation to mitigate the risks associated with general anesthesia, especially in older and frail populations.^2^, ^3^, ^4^, ^5^, ^6^ Conscious sedation allows for essential procedural guidance via transesophageal echocardiography (TEE) and helps to ensure patient stillness during the intervention, but discomfort and anxiety may increase procedural stress and complicate sedation management.^7^ Sedative agents required to ensure adequate procedural conditions may also contribute to cardiovascular, respiratory, and neurocognitive side effects in vulnerable patients. In this context, innovative nonpharmacological techniques for anxiety and sedation management are gaining attention. Audiovisual distraction (AVD) methods, such as virtual reality (VR) or video glasses, have been applied successfully across multiple surgical disciplines.^8^, ^9^, ^10^ In interventional cardiology procedures, VR use was associated with reduced sedation requirements without compromising procedural safety in transcatheter aortic valve replacement (TAVR).^11^ However, the application of AVD techniques in M-TEER remains unexplored, despite the increasing procedural volume and the growing need for patient-centered care strategies in this field of interventional cardiology. Given the unique challenges of M-TEER, including the need for intraprocedural echocardiographic imaging and prolonged procedural stillness, this study seeks to address this gap by evaluating the efficacy of AVD in reducing sedation requirements and enhancing patient comfort during M-TEER.

## Methods

### Study design

**Figure 1:**
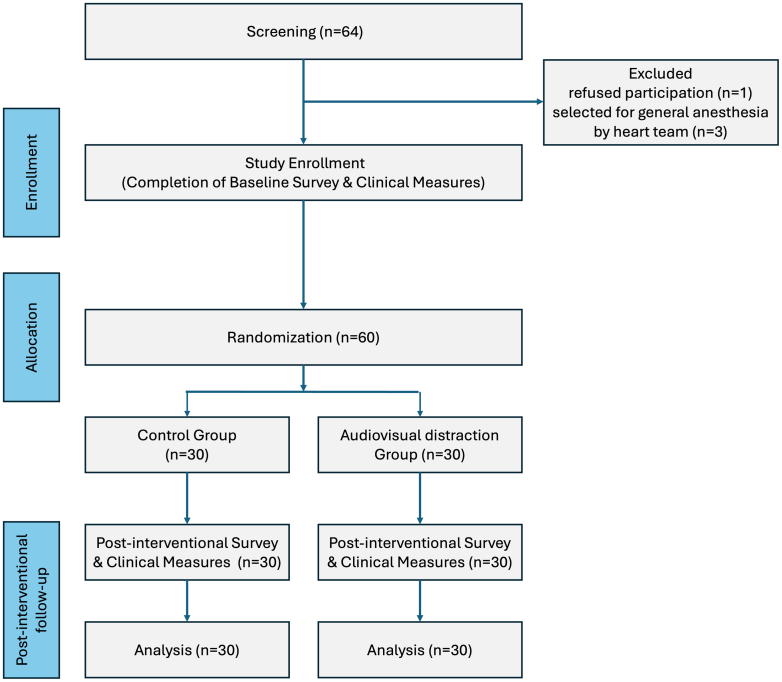
Study Flow A total of 64 patients scheduled for M-TEER under conscious sedation were screened. Four patients were excluded (1 refused participation and 3 were assigned to general anesthesia by the Heart Team), leaving 60 patients randomized 1:1 to sham control (inactive headset; n = 30) or active audiovisual distraction (n = 30). All randomized patients completed follow-up assessments and were included in the intention-to-treat analysis.

This study was designed as a single-center, prospective, randomized, sham-controlled trial. It was performed in accordance with the Declaration of Helsinki and institutional requirements. The study protocol was approved by the ethics committee of the University of Lübeck (AZ 21-306), and written informed consent was obtained from all participants before inclusion. The trial protocol was developed in accordance with the SPIRIT 2013 statement (Standard Protocol Items: Recommendations for Interventional Trials), and the reporting of this trial follows the CONSORT (Consolidated Standards of Reporting Trials) 2025 statement. The study is registered at the German Clinical Trials Register (DRKS00037370). The study flowchart is shown in Figure 1.

### Eligibility criteria

Adult patients with clinically relevant mitral regurgitation (MR) scheduled for M-TEER at the University Heart Center Lübeck were screened for eligibility.

Inclusion criteria were as follows: 1) indication for M-TEER as determined by the multidisciplinary Heart Team; 2) planned performance of the procedure under conscious or deep sedation rather than general anesthesia; and 3) ability to provide written informed consent and to complete the study questionnaires.

Exclusion criteria were barriers to effective communication (eg, language limitations or severe untreated hearing impairment), significant cognitive impairment or diagnosed dementia, known epilepsy or seizure disorder, uncorrectable visual deficits precluding the use of video glasses, severe chronic pulmonary disease, marked frailty or hemodynamic instability resulting in a Heart Team decision in favor of general anesthesia, and any other clinical reason requiring general anesthesia for the index M-TEER procedure.

### Sample size calculation

The sample size was determined a priori based on the expected difference in propofol dosage between groups. Assuming a clinically meaningful difference of 1.2 mg/kg/h, an SD of 1.5 mg/kg/h, a 2-sided alpha of 0.05, and 80% power, a 2-sample Student t-test yielded a required sample size of 26 patients per group (52 total). To account for possible dropout, incomplete questionnaire data, or possible conversion to general anesthesia, the planned enrollment was increased to 60 patients (30 per group). A total of 64 patients were screened; after application of the predefined exclusion criteria, 60 patients were randomized and included in the final analysis, consistent with the planned enrollment (Figure 1).

### Recruitment and randomization

Patients scheduled for M-TEER for MR at the University Heart Center Lübeck between February 2023 and November 2025 were assessed for eligibility according to the criteria described previously and were included after providing informed consent. Patients were evaluated preprocedurally by the multidisciplinary Heart Team, consisting of interventional cardiologists, cardiac surgeons, anesthesiologists, and imaging specialists. Patients were considered suitable for conscious sedation if they had preserved airway reflexes, stable hemodynamics, and no severe chronic pulmonary disease or marked frailty 2. In contrast, patients with a high risk for airway obstruction, severe frailty, or hemodynamic instability were considered unsuitable for conscious sedation and were assigned to general anesthesia according to Heart Team consensus. Patients selected for general anesthesia on this basis were excluded from the study.

Randomization was performed using a computer-generated list in a 1:1 ratio. Allocation concealment was ensured through sequentially numbered, opaque, sealed envelopes prepared by an independent research coordinator who was not involved in enrollment, device handling, or procedural management. After sedation had been initiated, the video-glasses system was either activated (AVD group) or left inactive (control group) according to the randomization. The anesthesiologist and anesthesia nurse responsible for sedation titration were blinded to the activation status. Because both groups wore identical headsets, the interventional and imaging teams were also blinded.

### Audiovisual distraction

**Figure 2:**
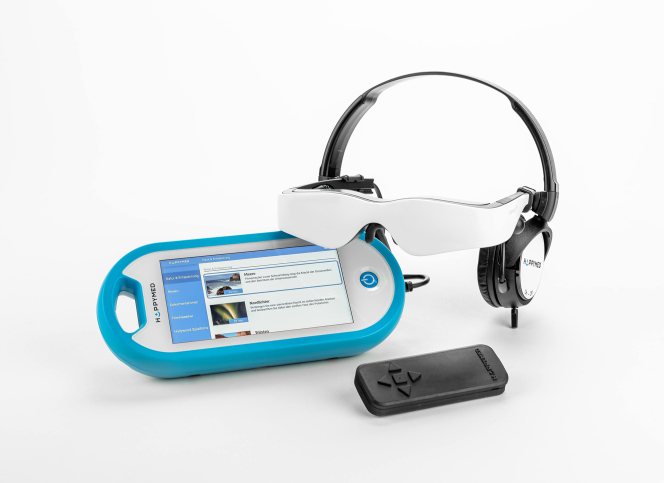
Headset Setup HappyMed video glasses and integrated headphones covered the patient's eyes and ears and delivered either active audiovisual content (AVD group) or an identical inactive sham device (control group).

AVD was implemented using HappyMed video glasses (HappyMed GmbH), a 2-dimensional entertainment video system equipped with integrated headphones. The design covers the patient's eyes and ears and creates an immersive audiovisual environment (Figure 2). The hardware is adjustable to ensure a comfortable and secure fit for individual patients. The video glasses are connected to a compact touchpad control panel that allows simple adjustments, including volume control.

In the control group, patients wore the same video glasses and headphones used in the intervention group, but the device remained inactive throughout the procedure. This sham-control approach provided a comparable physical sensation and partial sensory isolation without active audiovisual stimulation. Nature documentaries were selected as the content for the intervention, and patients could choose among mountain, desert, or ocean scenes.

### Periprocedural patient management

In both groups, patients received an initial intravenous bolus of 10 mg propofol, followed by a continuous infusion starting at 2 mg/kg/h. Sedation was titrated by the anesthesiologist or an experienced certified nurse, who was blinded to device activation, to achieve adequate calmness and immobility for TEE guidance and M-TEER. Sedation depth was assessed clinically using the Richmond Agitation-Sedation Scale (RASS). The RASS target was used pragmatically to harmonize sedation titration across both randomized groups. Propofol was adjusted to maintain a predefined target range of −2 to −3, corresponding to moderate procedural sedation with preserved spontaneous breathing and airway reflexes.

Because the video glasses covered the eyes, RASS scoring during headset use relied primarily on response to verbal command or light tactile stimulation, spontaneous movement, overall cooperation, respiratory pattern, and procedural tolerance of the TEE probe. If the RASS score was higher than −2, or cooperation/procedural tolerance of the TEE probe were inadequate, the propofol infusion rate was increased; if the RASS score was lower than −3, the infusion rate was reduced or temporarily interrupted. Details of the M-TEER procedure under deep sedation have been described previously.^2^

Norepinephrine was administered when necessary to maintain a target mean arterial pressure of at least 60 mm Hg. Before sedation, the video glasses and headphones were placed on the patient and the fit was checked. Depending on randomization, the system was then either activated or left inactive. Once the patient was adequately sedated, the TEE probe was inserted and the procedure began. In the AVD group, audiovisual content remained active throughout the procedure. At the end of the procedure, the propofol infusion was stopped, followed shortly by removal of the TEE probe. The video glasses remained in place until the patient had regained full consciousness and responsiveness and were then removed before transfer to the recovery room.

### Patient questionnaires

Before the intervention, participants were asked about their medical history and any hearing or visual impairments. Patients were also asked to complete preprocedural assessments for anxiety and pain levels using a numerical rating scale (NRS) (0 = no anxiety/pain, 10 = worst imaginable anxiety/pain) on the day of the procedure.

Postprocedurally, all patients completed a structured questionnaire evaluating their experience with the intervention. This included questions about anxiety and pain levels immediately after the procedure (NRS, 0-10). Additional questions addressed the comfort and usability of the video glasses, including whether the glasses caused any discomfort or pressure points, experience of nausea, and whether the video glasses helped improve their overall experience.

Two days after the procedure, all patients completed the German adaptation of the Client Satisfaction Questionnaire (ZUF-8), a validated instrument assessing overall satisfaction with inpatient management and care.^12^ This questionnaire captured a broader perspective on communication, management, and comfort during the hospital stay.

### Outcome definitions and delirium ascertainment

The primary endpoint was the mean propofol dose during the procedure (mg/kg/h). Secondary endpoints included norepinephrine dose, procedural duration, hemodynamic excursions, patient-reported anxiety and pain, overall satisfaction measured by ZUF-8, and in-hospital outcomes including intensive care unit stay, hospital stay, bleeding, conversion to general anesthesia, pneumonia, and delirium. Delirium was diagnosed based on the Diagnostic and Statistical Manual of Mental Disorders (DSM)-5 criteria.^13^

### Data availability statement

The data sets used and analyzed during the current study are available from the corresponding author on reasonable request.

### Statistical analysis

Continuous data are expressed as mean ± SD or mean ± 95% CI, as indicated. Data are presented as Tukey box-and-whisker plots (box = IQR, central line = median, whiskers = 1.5 × IQR; “+” indicates the mean). Normality of continuous variables was assessed using the Shapiro-Wilk test and visual inspection of histograms and quantile-quantile plots. For comparisons between independent groups, the Student *t*-test was used for normally distributed data, and the Mann-Whitney U test for non-normally distributed data. Differences between independent groups were calculated accordingly. For within- and between-group comparisons of anxiety and pain before and after the procedure, a 2-way analysis of variance with the factors group (AVD vs control) and time (before vs after procedure) was performed. Categorical data are expressed as counts and percentages. Chi-square tests were applied to compare categorical variables between groups, and the Wilcoxon rank test was used for paired ordinal data. All tests were 2-sided, and a *P* value <0.05 was considered statistically significant. Analyses followed the intention-to-treat principle. The specific statistical test used for each analysis is indicated in the corresponding table footnotes or figure legends. Prism (version 10.2.3, GraphPad Software) and SPSS (version 29.0, IBM Corp) were used for statistical analyses.

## Results

### Patient characteristics

Sixty-four patients were screened. One patient refused participation, and 3 patients had to be excluded as they were selected for general anesthesia based on the recommendations of the heart team. 30 patients were randomized to AVD group, 30 patients were randomized to control group. 51.7% of the 60 study patients were males with a mean age of 83.0 ± 6.3 years. The mean Society of Thoracic Surgeons score was 6.0 ± 3.9 which corresponds to a moderate-to-high risk patient population. There were no statistical differences between the AVD and the control group in the patients’ characteristics, comorbidities, and baseline echocardiographic parameters (Table 1).

**Table 1:** Baseline Characteristics

	Control (n = 30)	AVD (n = 30)	*P* Value
Age, y	82.1 ± 7.4	83.8 ± 4.9	0.30^a^
Female sex	46.7% (14/30)	50.0% (15/30)	1.00
Body mass index, kg/m^2^	25.3 ± 5.6	25.2 ± 5.9	0.91^a^
NYHA functional class	2.8 ± 0.7	2.7 ± 0.6	0.51^b^
Diabetes	13.3% (4/30)	6.7% (2/30)	0.67
Hypertension	56.7% (17/30)	56.7% (17/30)	1.00
Coronary artery disease	33.3% (10/30)	36.7% (11/30)	1.00
COPD	6.7% (2/30)	6.7% (2/30)	1.00
Estimated GFR, mL/min/1.73 m^2^	49.9 ± 18.2	51.2 ± 19.1	0.78^a^
LV ejection fraction, %	48.6 ± 9.9	49.8 ± 7.9	0.61^a^
MR grade	3.4 ± 0.5	3.3 ± 0.5	0.44^b^
STS score	5.8 ± 4.0	6.2 ± 3.9	0.70^a^

AVD = audiovisual distraction; COPD = chronic obstructive pulmonary disease; GFR = glomerular filtration rate; LV = left ventricular; MR = mitral regurgitation; STS = Society of Thoracic Surgeons.

a *P* values were calculated using Student *t*-tests for normally distributed continuous variables.

b Mann-Whitney U tests for ordinal or non-normally distributed variables and Fisher exact tests for categorical variables.

A total of 35 patients underwent MitraClip (Abbott) implantation, whereas 22 patients received a Pascal device (Edwards Lifesciences). In 2 cases, no device was implanted due to the development of a transvalvular gradient following provisional device placement. In 1 case, the procedure was aborted because a safe trans-septal puncture could not be performed due to anatomical reasons. In the AVD group, 1 patient required conversion to general anesthesia and intubation due to respiratory insufficiency. One patient in the control group required general anesthesia and intubation due to significant oral bleeding during the procedure. Following the principles of an intention-to-treat analysis, the data from these patients were included in all subsequent analyses.

### Peri-interventional drug treatment and procedural time

**Figure 3:**
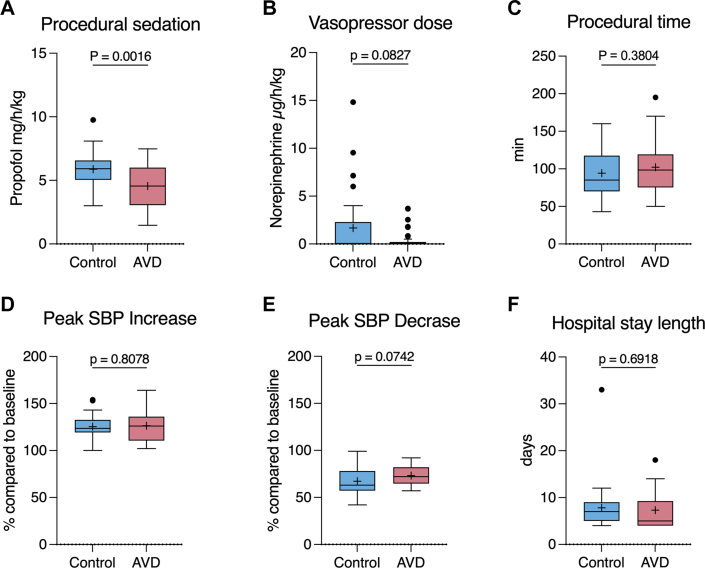
Sedation and Procedural Metrics (A) Propofol dose, (B) norepinephrine dose, (C) procedural time, (D) peak systolic blood pressure (SBP) increase and (E) decrease, and (F) hospital length of stay are shown for both groups. The AVD group required significantly less propofol, whereas norepinephrine use, hemodynamic excursions, procedural time, and length of stay were not significantly different. Data are displayed as Tukey box-and-whisker plots; exact *P* values are shown in the figure panels. Propofol dose (A), procedural time (C), and SBP excursions (D and E) were analyzed using unpaired Student t tests; norepinephrine dose (B) and length of stay were analyzed using Mann-Whitney U tests. AVD = audiovisual distraction.

The mean propofol dosage was significantly lower in the AVD group compared to the control group (4.6 ± 1.6 mg/kg/h vs 5.9% ± 1.4 mg/kg/h; *P* = 0.002) (Figure 3A). Norepinephrine dose was numerically lower in the AVD group, but the difference was not statistically significant (AVD: 0.5 ± 1.1 μg/kg/h vs control group: 1.7 ± 3.4 μg/kg/h; *P* = 0.08) (Figure 3B). Procedural time, defined as the duration from the start of sedation to the removal of the TEE probe, did not differ significantly between the groups (AVD: 102.2 ± 36.0 minutes vs control group: 94.2 ± 32.4 minutes; *P* = 0.38) (Figure 3C).

### Periprocedural hemodynamics

The maximum relative increase in systolic blood pressure was not significantly different between groups (126.4% ± 16.5% in the AVD group vs 125.4% ± 14.0% in the control group; *P* = 0.81) (Figure 3D). The same applied to the maximum relative decrease in systolic blood pressure (73.2% ± 9.8% in the AVD group vs 67.2% ± 15.2% in the control group; *P* = 0.074) (Figure 3E).

### Procedural outcomes

Regarding procedural success, the average reduction in MR grade was 2.6 ± 0.1, with no significant difference between groups (Table 2). Final residual MR was ≤1+ in 90% of patients in both groups. The mean length of hospital stay was 7.5 ± 4.7 days, with no statistically significant difference between the groups (7.3 ± 3.9 days in the AVD group vs 7.8 ± 5.7 days in the control group; *P* = 0.69) (Figure 3F). Delirium was reported in 1 patient from the control group and none in the AVD group. There were no significant group differences regarding the incidence of both major and minor bleeding as well as postinterventional pneumonia (Table 2). No patient died during the hospital stay.

**Table 2:** Procedural Outcomes

	Control (n = 30)	AVD (n = 30)	*P* Value
MR grade reduction	2.6 ± 0.1	2.6 ± 0.1	1.00^a^
Length of hospital stay, days	7.8 ± 5.7	7.3 ± 3.9	0.69^a^
Length of ICU stay, days	0.9 ± 3.6	0.2 ± 0.5	0.28^a^
Major bleeding (BARC ≥3)	3.3% (1/30)	0% (0/30)	1.00
Minor bleeding (BARC ≤2)	13.3% (4/30)	16.7% (5/30)	1.00
Conversion to general anesthesia	3.3% (1/30)	3.3% (1/30)	1.00
Clinically diagnosed delirium	3.3% (1/30)	0% (0/30)	1.00
Postinterventional pneumonia	3.3% (1/30)	0% (0/30)	1.00

BARC = Bleeding Academic Research Consortium; ICU = intensive care unit; other abbreviations as in Table 1.

a *P* values were calculated using Mann-Whitney U tests for ordinal or non-normally distributed variables and Fisher exact tests for categorical variables.

### Patient comfort and satisfaction

**Figure 4:**
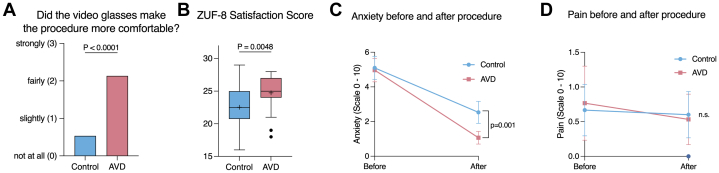
Patient-Reported Outcomes The AVD group reported (A) higher comfort scores, (B) greater overall satisfaction on the ZUF-8 questionnaire, and (C) lower postprocedural anxiety than the control group. (D) Pain scores remained low in both groups before and after the procedure and did not differ significantly. Comfort scores (A) were analyzed using a Mann-Whitney U test, ZUF-8 scores (B) using an unpaired Student t test, and anxiety and pain trajectories (C and D) using 2-way ANOVA with factors group and time. ZUF-8 = German Patient Satisfaction Questionnaire; other abbreviation as in Figure 3.

On a 4-point scale ranging from 0 to 3, patients in the AVD group reported significantly higher scores to the question of whether the video glasses made the procedure more comfortable (Figure 4A). Consistently, overall patient satisfaction with the hospital stay, as measured by the ZUF-8 score, was significantly higher in the AVD group than in the control group (24.7 ± 3.0 vs 22.5 ± 2.8; *P* = 0.005) (Figure 4B).

### Periprocedural anxiety, pain, and nausea

Results on periprocedural anxiety are illustrated in Figures 4C and 4D. On average, patients in both groups reported moderate levels of anxiety before the M-TEER procedure, with no significant difference observed (NRS 5.1 ± 1.8 in the AVD vs 5.1 ± 1.9 in the control group; *P* = 0.93). However, immediately postintervention, patients in the AVD reported significantly lower anxiety levels compared to the control group (NRS 1.1 ± 1.0 vs 2.5 ± 1.7; *P* = 0.001). In contrast, subjective pain levels were consistently low in both groups, with no significant differences preintervention or postintervention. Preintervention pain scores were NRS 0.77 ± 0.5 in the AVD vs 0.67 ± 1.0 in the control group (*P* = 0.91), whereas postintervention scores were NRS 0.53 ± 1.0 vs 0.60 ± 0.9 (*P* = 0.90). Nausea after the procedure was reported in 2 patients from the AVD and 1 patient from the control group (*P* = 0.59).

## Discussion

In this randomized controlled trial of patients undergoing M-TEER under conscious sedation, AVD with video glasses reduced propofol requirements and improved patient-reported comfort, anxiety, and satisfaction, with similar procedural time, hemodynamic excursions, and in-hospital safety compared with sham control (Central Illustration). These findings support AVD as a simple, nonpharmacological adjunct to contemporary minimalist strategies in structural heart interventions and extend prior evidence on VR- and AVD-based approaches from other interventional settings to the specific context of M-TEER.^7^

**Central Illustration:**
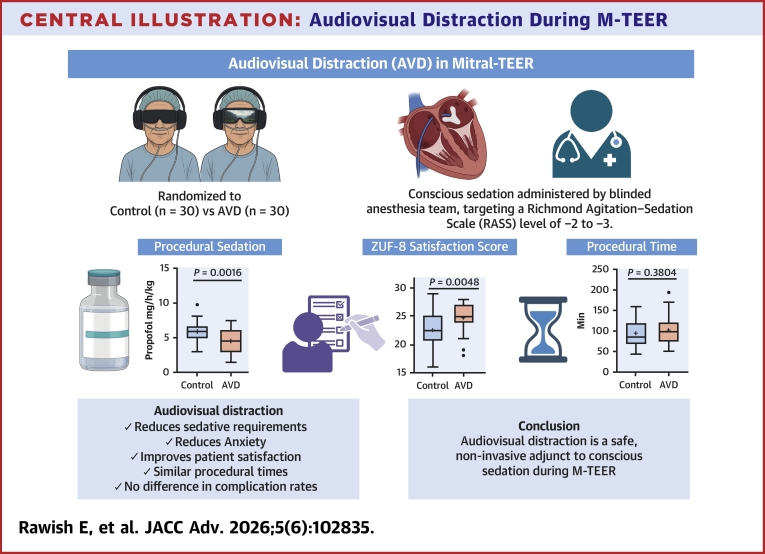
Audiovisual Distraction During M-TEER Patients undergoing M-TEER under conscious sedation were randomized 1:1 to active audiovisual distraction or sham control with an identical inactive device. Active audiovisual distraction reduced propofol dose and anxiety, improved satisfaction, and was associated with similar procedural time and complication rates. AVD = audiovisual distraction; M-TEER = mitral transcatheter edge-to-edge repair; ZUF-8 = German Patient Satisfaction Questionnaire.

The choice of anesthetic strategy has become a central component of M-TEER programs. Deep conscious sedation has emerged as a safe and efficient alternative to general anesthesia, with meta-analyses and individual-patient-data analyses demonstrating comparable procedural success and mortality, but shorter length of stay and fewer resource demands.^6^

In this context, the need for continuous TEE guidance and prolonged procedural stillness often drives relatively high sedative doses, especially in very old and frail patients. Our data show that AVD can attenuate this pharmacological burden: propofol infusion rates were reduced by approximately 1.3 mg/kg/h in the AVD group, whereas procedural time, technical success, and residual MR were indistinguishable from the control group. This suggests that AVD enables operators to maintain procedural quality and imaging conditions while operating at a lighter level of pharmacological sedation. The clinical importance of sedation sparing in this very elderly cohort should be interpreted carefully. Our trial was powered for propofol dose, not for hard clinical events. Accordingly, we did not observe significant between-group differences in bleeding, pneumonia, intensive care unit stay, hospital stay, conversion to general anesthesia, or delirium.

The numerically lower norepinephrine use is hypothesis-generating only. Given the known associations between deeper or prolonged sedation and hypotension, respiratory depression, delayed recovery, and delirium in older adults, even modest reductions in sedative burden may be clinically relevant and should be tested in larger trials with adequate power and structured recovery endpoints.^14^, ^15^, ^16^, ^17^ Thus, further studies with more patients, structured recovery, neurocognitive, and resource-utilization endpoints are needed to determine whether sedative sparing translates into clinically measurable outcome benefit.

Beyond pharmacological sparing, our findings underscore the value of systematically integrating patient-centered outcomes into the evaluation of structural heart interventions. Patients randomized to AVD reported higher intraprocedural comfort and significantly greater overall satisfaction with their hospital stay, as reflected by the ZUF-8 score. These results mirror prior work in interventional cardiology and interventional radiology, in which VR or video-glasses-based distraction consistently improved patient experience and reduced periprocedural anxiety and distress.^18^, ^19^, ^20^

Importantly, to our knowledge, this is the first randomized trial to evaluate AVD in the setting of M-TEER. In our study, preprocedural anxiety levels were comparable between groups, but postprocedural anxiety was significantly lower in the AVD group, suggesting that immersive distraction during the procedure has a sustained effect on how patients retrospectively appraise their experience. This aligns with the concept, emphasized recently, that managing interventional anxiety is an emerging quality metric in interventional cardiology, particularly as procedures are increasingly performed under local anesthesia or light sedation.^7^

An important design feature of our trial is the use of an identical but inactive headset in the control arm. This sham-control approach was chosen to preserve blinding of sedation management and to isolate the incremental effect of active audiovisual content beyond simply wearing the device. At the same time, this design does not fully disentangle the effects of active distraction from partial visual shielding or environmental attenuation. Some of the observed benefit may therefore reflect a composite of active audiovisual stimulation and nonspecific sensory isolation. Future three-arm studies comparing active AVD, sham headset, and standard care without a headset would be valuable.

From an implementation standpoint, the present data support AVD as a pragmatic tool that can be integrated into standard cath lab and hybrid operating room workflows without prolonging procedures or increasing staff workload. The AVD system used in our trial is 2-dimensional and relatively low-immersive compared with fully immersive VR headsets, which may explain the very low incidence of nausea and absence of relevant cybersickness in both arms.^21^^,^^22^ This is an important consideration for M-TEER, where the patient must tolerate a TEE probe and supine positioning over an extended period. The ability to offer a well-tolerated, standardized nonpharmacological adjunct that improves patient experience and reduces sedative dose, without adding complexity or time, is attractive for centers scaling up M-TEER programs and striving for streamlined, high-throughput pathways.

Our findings also complement and extend prior work on AVD and VR in TAVR. Randomized pilot studies have demonstrated that VR or AVD during TAVR under conscious sedation is feasible and safe and may reduce periprocedural anxiety; some, but not all, studies reported improvements in pain and sedative requirements.^11^^,^^19^, ^20^, ^21^

In contrast to TAVR, M-TEER typically involves longer procedure times and continuous TEE guidance, with a greater need for patient immobility. Demonstrating that AVD remains effective and well tolerated under these conditions is therefore an important step towards broader adoption of nonpharmacological adjuncts across the spectrum of transcatheter valve interventions. Future work may explore whether combining AVD with protocolized lighter sedation and objective sedation-depth monitoring can further optimize outcomes and resource utilization in structural heart disease programs.

### Study Limitations

Several limitations merit consideration. First, this was a single-center trial with a modest sample size, which limits the precision of estimates for secondary endpoints and rare adverse events. Although the patient cohort reflects a typical elderly, comorbid M-TEER population, external validity to other centers with different sedation protocols, case mix or organizational structures remains to be established. Second, all patients were preselected by a Heart Team as suitable for M-TEER under conscious sedation; individuals deemed to require general anesthesia were excluded. Consequently, our findings cannot be extrapolated to the sickest or most unstable patients, in whom the risk-benefit profile of AVD may differ. Third, RASS was used pragmatically to harmonize sedation titration, but it has not been specifically validated for anesthesia-administered sedation during M-TEER or for patients wearing video glasses. Alternative responsiveness-based scales such as the Modified Observer's Assessment of Alertness/Sedation^23^ or the Extended Observer's Assessment of Alertness/Sedation^24^ may be better suited for future studies. Because both groups were managed by the same blinded anesthesia team using the same target range, this limitation is likely nondifferential, but residual variability in true sedation depth cannot be excluded. We did not use processed electroencephalographic monitoring; however, its added value for differentiating lighter levels of sedation remains uncertain. Fourth, we also did not formally measure how continuously patients attended to the video content. At the stated target RASS, the intervention should be viewed less as sustained active viewing throughout every minute of the procedure and more as a calming sensory frame that may be most relevant during induction, TEE insertion, and other stressful procedural phases. Fifth, delirium was captured clinically by DSM-5 criteria,^13^ but not assessed with a structured delirium tool; therefore, our study is not able to address the effect of AVD-mediated sedative sparing on postoperative delirium or longer-term cognitive function. Finally, although we observed significant improvements in periprocedural comfort, anxiety and overall satisfaction, long-term quality-of-life and functional outcomes need to be assessed by larger future trials.

## Conclusions

In summary, this randomized controlled trial shows that AVD using video glasses is a feasible and well-tolerated adjunct to conscious sedation in patients undergoing M-TEER. AVD significantly reduces propofol requirements and improves patient comfort and satisfaction without prolonging procedures or compromising hemodynamic stability and procedural outcomes. These data support the integration of AVD into patient-centered, minimalist M-TEER pathways and provide a strong rationale for larger, multicenter studies powered for hard clinical, cognitive, and economic endpoints.

Perspectives **COMPETENCY IN MEDICAL KNOWLEDGE:** AVD represents a nonpharmacological adjunct to conscious sedation in M-TEER. In this randomized trial, AVD significantly reduced propofol requirements under a uniform blinded sedation protocol, with similar procedural metrics and in-hospital safety compared with sham control.

**COMPETENCY IN PATIENT CARE:** The integration of AVD into M-TEER procedures can improve patient-reported outcomes, including procedural comfort, anxiety, and overall satisfaction, without prolonging procedural time or increasing complications. This approach supports a patient-centered, minimalist strategy that can reduce sedative exposure in elderly and frail patients while maintaining optimal procedural conditions.

**TRANSLATIONAL OUTLOOK:** Future larger multicenter studies are warranted to evaluate the impact of AVD-guided sedation strategies on clinically relevant outcomes, including delirium, cognitive recovery, and resource utilization. Future protocols should incorporate validated procedural sedation scales and structured recovery assessments.

## Funding support and author disclosures

Dr Rawish is supported by the 10.13039/100010447German Centre for Cardiovascular Research (DZHK Clinician Scientist Program, 81X3700107). Dr Rawish and Dr Eitel are supported by the 10.13039/100018693Horizon Europe Program (Grant No. 101156555). Dr Stiermaier reports lecture honoraria and travel support from 10.13039/100011949Abbott Vascular. Dr Eitel reports lecture honoraria and travel support from 10.13039/100011949Abbott Vascular and Edwards Lifesciences. Dr Frerker reports lecture honoraria, travel support, and research grants from 10.13039/100011949Abbott Vascular and Edwards Lifesciences. Dr Schmidt reports lecture honoraria and travel support from Abbott Vascular and Edwards Lifesciences. All other authors have reported that they have no relationships relevant to the contents of this paper to disclose.
